# Molecular Neurobiology and Promising New Treatment in Depression

**DOI:** 10.3390/ijms17030381

**Published:** 2016-03-15

**Authors:** Sang Won Jeon, Yong-Ku Kim

**Affiliations:** Department of Psychiatry, College of Medicine, Korea University, Ansan Hospital, 123, Jeokgeum-ro, Danwon-gu, Ansan-si, Gyeonggi-do, Seoul 15355, Korea; npdrjeon@korea.ac.kr

**Keywords:** depression, neurobiology, antidepressant, neural plasticity, BDNF, endocrine, immune, gene, mTOR

## Abstract

The limited effects of currently available antidepressants are becoming an urgent issue in depression research. It takes a long time to determine treatment effects, and the overall remission rate is low. Although we expect the development of non-monoamine antidepressants in the near future, efforts in this regard over the past several decades have not yet been compensated. Thus, researchers and clinicians should clarify the neurobiological mechanisms of integrated modulators that regulate changes in genes, cells, the brain, and behaviors associated with depression. In this study, we review molecular neurobiological theories and new treatments for depression. Beyond neuroanatomy and monoamine theory, we discuss cells and molecules, neural plasticity, neurotrophisms, endocrine mechanisms, immunological mechanisms, genetics, circadian rhythms, and metabolic regulation in depression. In addition, we introduce the possibility of new antidepressant drug development using protein translation signaling (mTOR) pathways.

## 1. Introduction

Depression is currently one of the most important causes of mortality and morbidity, which occurs in all genders, ages, and social backgrounds. Serious problems, such as suicidal behavior, is frequently occurring in the patients with depression. Although many psychopharmacological agents are currently available for the treatment of depression, a relevant percentage of patients (20%–30%) treated with commonly used antidepressants do not achieve complete recovery and develop a treatment-resistant depression [1]. The most important reason associated with this disabling disorder is that depression is a multifaceted disorder with diverse causes and that our knowledge about its pathogenetic mechanisms is limited [2].

The pathogenesis and treatment mechanisms of depression are now understood thanks to multilateral neurobiological studies and the introduction and development of antidepressants. The importance of the neurotransmission system in patients with depression has been recognized alongside the introduction of antidepressants. The structural and functional brain anomalies of patients with depression have been investigated through brain imaging studies. In addition, genetic mutations have been reported through genetic studies. Molecular biological studies and gene manipulation tests have also contributed to mounting evidence regarding the preclinical grounds associated with depression.

The monoamine neurotransmission system, which includes serotonin and norepinephrine systems is the primary target of most major antidepressant drugs that are currently used. Drugs that increase serotonin and norepinephrine levels at synaptic junctions through reuptake inhibition were developed first, followed by drugs that affect dopamine and acetylcholine systems. Most recently, the rapid antidepressant effects of a non-competitive NMDA (N-methyl-D-aspartic acid) glutamate receptor antagonist have been reported [3]. Various neurotransmission systems must be considered in order to develop new antidepressants, which typically require a common molecular pathway that involves more than a mere neurotransmitter receptor.

Our understanding of the neurobiological mechanisms of depression is still poor, and the therapeutic effects of antidepressants are limited. It takes a long time to obtain treatment effects, and the overall remission rate is low. Neurogenic mechanisms, which are thought to play a key role in antidepressant action, can explain only part of the current treatment mechanisms for depression. Thus, it is necessary to clarify the neurobiological mechanisms of the integrated major modulators that regulate changes in the genes, cells, the brain, and in behaviors associated with depression.

In this study, we review molecular neurobiological theories and new treatments for depression. We specifically discuss neural circuitry, the monoamine hypothesis, neural plasticity, the neurotrophic and brain-derived neurotrophic factor (BDNF) hypothesis, neuroendocrine mechanisms, the neuroimmune and cytokine hypothesis, and genetic biomarkers. In addition, we introduce the possibility of new antidepressant drug development using protein translation signaling (mTOR) pathways.

## 2. Results and Discussion

### 2.1. Neural Circuitry

According to previous studies, the limbic-cortical-striatal-pallidal-thalamic tract, which is connected to the hippocampus, amygdala, caudate nucleus, putamen, and the frontal cortex, is thought to be the key neuroanatomical circuit in depression [4].

The hippocampus is the most widely studied brain structure associated with stress, depression, and antidepressant mechanisms. Since the hippocampus is associated with learning and memory, it is also associated with cognitive impairment in depression. The hippocampus is involved in the feedback loop of the hypothalamic-pituitary-adrenal (HPA) axis, and this can result in neuroendocrine disorders [5]. Chronic stress induces atrophic changes in hippocampal subregions, and hippocampal volume decreases in depression [6].

Deterioration of prefrontal cortex (PFC) functions can cause various symptoms such as depressive mood, working memory impairment, and psychomotor retardation, because the PFC is neurally connected to the hippocampus, basal ganglia, thalamus, ventral tegmental area, dorsal raphe nucleus, and locus ceruleus, which are closely associated with the pathophysiology of depression [7].

The amygdala allocates or assigns emotional importance upon receiving psychological stimuli. In addition, the amygdala is associated with the pharmacological actions of antidepressant treatments [8]. A decrease in the amygdala volume [9] and a loss of glial cells [10] have been reported in patients suffering from depression.

The mesolimbic dopamine system is involved in motivation and reward mechanisms, and is neurally connected from the ventral tegmental area (VTA) to the nucleus accumbens (NAc). This system is specifically associated with the anhedonia and anergy of depression [11]. Antidepressant treatments increase dopamine neural transmission in the nucleus accumbens [12].

The hypothalamus is thought to be significantly associated with the neurophysiological symptoms of depression, including insomnia, loss of appetite, and loss of sexual desires. In summary, multiple brain regions are probably associated with the pathophysiology of depression. Dysfunction can separately develop in multiple brain regions, and dysfunction in a specific region can cause damage to other regions.

### 2.2. The Monoamine Hypothesis and Its Limitations

The monoamine hypothesis has long been recognized as a core concept in the pathogenesis of depression. According to this hypothesis, depression can be attributed to the functional imbalance or deficiency of monoamine-series neurotransmitters, including dopamine, serotonin (5-HT), and norepinephrine (NE). The hypothesis is supported by the antidepressant effects of tricyclics and monoamine oxidase inhibitors, which were accidentally discovered. These effects enhance short-term monoamine functions by inhibiting the function of the monoamine transporters. The introduction of selective serotonin reuptake inhibitors (SSRI) provided further support for this hypothesis. Based on these concepts, the monoamine depletion hypothesis was suggested [13]. According to this hypothesis, the depletion of serotonin and norepinephrine in the synapse triggers the development of depressive symptoms. However, this hypothesis cannot explain the three to four weeks required to obtain treatment responses, even though monoamine levels sharply increase upon antidepressant administration.

The monoamine receptor sensitivity hypothesis was suggested to address this limitation [14]. According to this hypothesis, monoamines are continuously secreted or increased by the administration of an antidepressant, and the receptors at the pre- and post-synapse are activated. As a results, the number of monoamine receptors decreases (down-regulation), showing treatment effects. In other words, depression is induced by monoamine receptor up-regulation. The long-term administration of antidepressants results in a down-regulation of the β-adrenergic receptor (βAR) ligand binding site in the limbic system, including in the brain cortex and hippocampus [15]. In addition, antidepressants down-regulated the 5-HT2A receptor in the rat brain [16]. However, this hypothesis also has some limitations. First, not all antidepressants down-regulate the βAR or 5-HT2A ligand binding site. Second, down-regulation of the βAR or 5-HT2A binding site occurs before treatment reactions appear [17].

The 5-HT1A receptor sensitivity hypothesis was suggested to explain the time gap [18]. According to this hypothesis, depression develops due to abnormally increased somatodendritic 5-HT1A autoreceptor function, and antidepressants work by down-regulating the presynaptic 5-HT1A receptor. 5-HT reuptake inhibition by SSRI increases the 5-HT level in the synapse upon completion of the adaptive desensitization of the presynaptic 5-HT1A receptor.

Therefore, it may not be appropriate to explain depression using a simple monoamine deficiency model. Although antidepressants based on the monoamine hypothesis are still the first choice for treating depression, it takes three to four weeks to obtain treatment effects, and remission is uncommon. For these reasons, more effective antidepressants are required.

### 2.3. Neural Plasticity and Neurogenesis

In molecular and cellular theory, stress, depression, and the mechanism of antidepressant action are explained using the concept of an intracellular signal transduction cascade and neural plasticity. According to this hypothesis, stress-induced dysfunction of signal-transduction-cascades and the failure of neuronal adaptation in specific brain regions induce stress-related diseases, and antidepressants ameliorate these problems. Severe stress causes hippocampal neuronal cell atrophy and death and also inhibits neurogenesis, resulting in structural changes and functional impairment of the hippocampus. Depression develops due to a similar mechanism.

**Figure 1:**
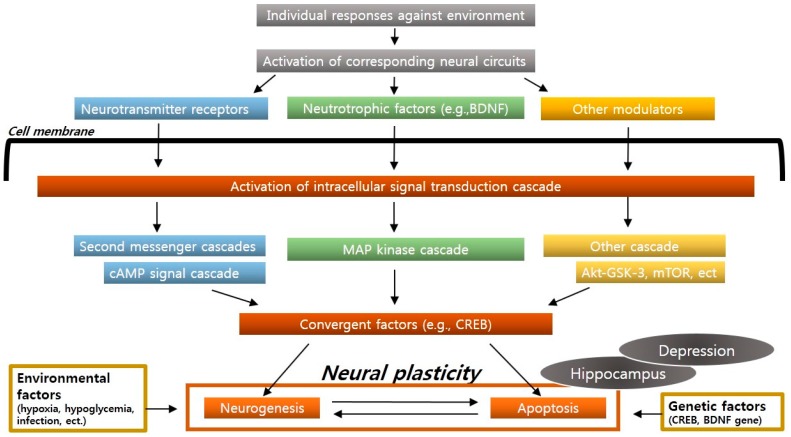
The concept of an intracellular signal transduction cascade and neural plasticity.

Neural plasticity or structural plasticity is defined as a neuronal adaptation that is an individual response to the environment, which includes new cell formation and genetically healthy cell death in the adult brain [19]. The former is called neurogenesis, and the latter, apoptosis. Each specific neural circuit is activated by learning, memory, stress, or the environment, which induce an intracellular signal transduction cascade that is a core function of neural plasticity. The known circuits include a cyclic adenosine monophosphate (cAMP) signal transduction cascade and a neurotrophic factor signal transduction cascade. These cascades are gradually collected by convergent factors (e.g., cyclic adenosine monophosphate response element-binding protein (CREB)) [20]. Molecular or cellular adaptations for neural plasticity, including cell survival or death processes, occur through these convergent factors. The signal transduction cascade plays a key role in regulating neuronal atrophy, neuronal death, and neurogenesis, which occur due to stress and depression. Environmental factors (such as hypoxia, hypoglycemia, neural toxicity, and viral infection) or genetic factors (*CREB* or *BDNF* gene expression changes or mutations) that inhibit neuronal functions and activities promote neuronal atrophy or death (Figure 1).

Each specific neural circuit is activated by learning, memory, stress, or the environment, which induces appropriate intracellular signal transduction cascades. These cascades are gradually collected into convergent factors (CREB), which promote neural plasticity, including cell survival or death. Imbalances in neural plasticity, particularly in the hippocampus, cause depression.

In summary, signal transduction cascades and neuronal adaptation are very important for the pathogenesis of depression, and their dysfunction or failure causes stress-related diseases such as depression. Antidepressants help patients recover from these problems.

### 2.4. The Neurotrophic and Brain-Derived Neurotrophic Factor (BDNF) Hypothesis

Previous reports have demonstrated decreased hippocampal and prefrontal cortex volume in patients with depression [6]. This supports the hypothesis that the expression of nerve growth factors, specifically BDNF, is reduced in patients with depression. BDNF is frequently expressed in the adult limbic system. The BDNF hypothesis is that depression is based on neurotrophin deficiency in the hippocampus, and that antidepressants normalize this deficiency [21,22]. This BDNF hypothesis is based on the adverse effects of stress on the hippocampus. Long- and short-term stress reduces BDNF expression in the hippocampus, whereas long-term administration of antidepressants shows opposite results, increasing resistance to stress. Accordingly, improvement in BDNF function can contribute to recovery of nerve cells in the hippocampus from stress-induced damage, and may confer protection against further damage. BDNF is also known to increase synapse plasticity in the hippocampus [23] (Figure 1). Thus, long-term treatment with antidepressants may improve overall hippocampal function. The BDNF neurotrophic hypothesis can explain the reason that long-term antidepressant administration is required to obtain treatment effects.

BDNF changes were observed in postmortem biopsies of hippocampi from depressed patients, as were changes in plasma BDNF concentrations. However, recent studies demonstrate that this hypothesis requires further refinement. First, BDNF changes in response to stress and antidepressants have not been verified, and contrary results have been reported [24]. Second, removal of BDNF or BDNF receptors from the frontal lobe of male mice did not produce behavior similar to depression [25]. Lastly, the increased BDNF in the VTA and NAc somewhat induced depression [26].

**Figure 2:**
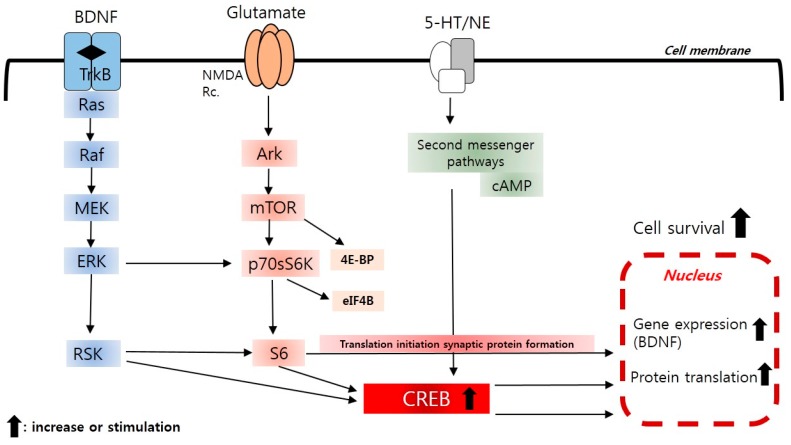
Neurotrophic factor (mitogen activated protein (MAP) kinase cascades; e.g., brain-derived neurotrophic factor (BDNF)) and protein translation signal pathways (protein translation signaling (mTOR); glutamate).

Although the neurotrophic hypothesis has not been verified, it has made BDNF and the BDNF receptor (TrkB) a focus of antidepressant targets (Figure 2). Theoretically, the direct antagonism of TrkB and many downstream factors can have antidepressant action, but the development of therapeutic agents is challenging. The reasons are as follows. First, BDNF is an approximately 14-kDa protein that forms a dimer to function on the TrkB receptors. Therefore, it is technically difficult to develop small molecules against the TrkB receptor. Second, the TrkB downstream signal transduction cascades include the Ras-Raf-ERK, PLCγ, and PI3K-Akt cascades [27]. It remains uncertain which of these cascades is closely associated with antidepressant effects. Third, these signal transduction cascades are involved in many essential physiological functions in the brain, so the simple antagonism of TrkB is likely to be severely toxic. Fourth, the antidepressant properties of BDNF may be circuitry-specific, so BDNF may aggravate depressive symptoms by acting within a specific circuit, such as the ventral tegmental area (VTA) → the nucleus accumbens (NAc) tract, rather than within the entire hippocampus [21]. In other words, BDNF antagonism can induce very complicated results. Nonetheless, BDNF neurotrophism remains a potential target for the development of new antidepressants.

A neurotrophic factor signal pathway shows how BDNF increases neurogenesis in the hippocampus. Active protein translation processes are required for neurogenesis. Protein translation through mTOR cascades is regulated by a dynamically-reciprocal signal transmission system. NMDA receptor antagonists may exert their antidepressant effects through protection of nerve cells and inhibition of nerve cell damage caused by an increase in NMDA receptor hyperactivity.

### 2.5. Neuroendocrine Mechanisms

**Figure 3:**
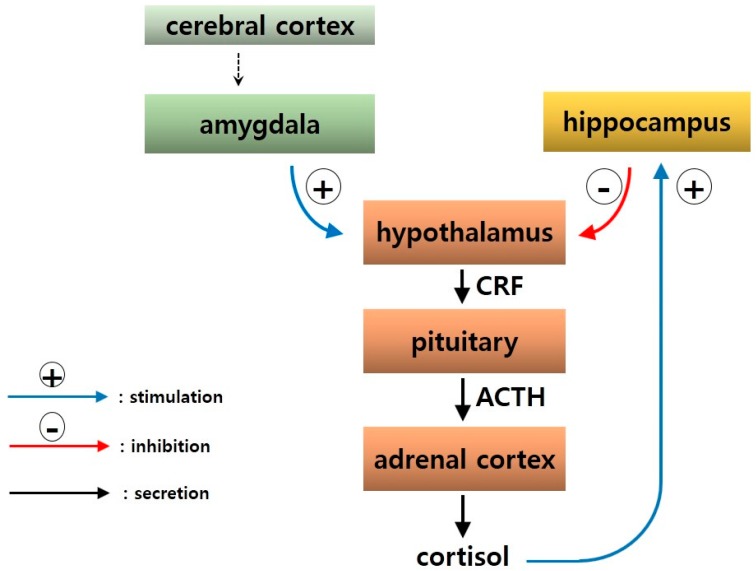
Neural circuitry of the hypothalamic-pituitary-adrenal (HPA) axis as a feedback loop.

Stress may not be sufficient to induce depression, but is an important factor in the pathogenesis of depression. Thus, the HPA axis, which is an important factor in resisting stress, has attracted considerable scientific attention. Corticotropin-releasing factor (CRF), which is secreted from the paraventricular nucleus (PVN) of the hypothalamus, increases adrenocorticotropin (ACTH) secretion from the pituitary. As a result, glucocorticoid (human cortisol) is secreted from the adrenal cortex and affects neurobehavioral functions in many brain domains. The HPA axis forms a feedback loop with brain domains such as the hippocampus and amygdala [28] (Figure 3).

Damage in the hippocampal CA3 pyramidal neurons occurs when severe stress continues for a long time and a high blood concentration of glucocorticoids is sustained [6,29]. Hypercortisolemia increases excitotoxicity of the pyramidal neurons in the hippocampus and eventually causes dendritic atrophy, spinal reduction, and even nerve cell death in extreme cases. Additionally, new nerve cell formation in the granule layer is inhibited. These functional problems in the hippocampus caused by long-term stress can deteriorate the inhibitory tone that the hippocampus gives to the HPA axis. Consequently, the HPA axis can be excessively activated. This excessive activity is observed in approximately 50% of depressed patients, and the continuous administration of antidepressants is known to relieve these phenomena [30]. In addition, continuous antidepressant treatment increased new neuronal formation and reversed the aforementioned abnormal hippocampal functions.

From this perspective, an intentional weakening of CRF function to enhance resistance to hypercortisolemia was suggested as a depression treatment [31]. It remains unclear whether weakening the hyperactive HPA axis can ameliorate depression. One of the representative targets is the CRF receptors (CRF1 (predominant) and CRF2), as is it unknown whether their antagonists have antidepressant properties. Positive therapeutic effects of CRF1 antagonist were reported in animal experiments [32], but no consistent outcomes have been reported in humans. One open label clinical trial reported that a CRF1 antagonist reduced depressive symptoms in depressed patients, without interference of the HPA axis [33]. However, no well-controlled study has verified these finding. There are some issues in the development of CRF1 receptor antagonists. Pharmacokinetic properties (excessively lipophilic and poor aqueous solubility) and hepatotoxicity issues have led to the discontinuation of CRF1 antagonists [34].

In the meantime, the vasopressin receptor is drawing attention. Vasopressin is a neuropeptide that reinforces CRF function. It functions through vasopressin receptors that are expressed in the amygdala and limbic system. Vasopressin receptor antagonists have shown antidepressant effects through the amygdala in experimental animals, but not in humans [35].

Direct antagonism against the glucocorticoid receptor has also attracted attention. The glucocorticoid receptor antagonist mifepristone (RU486) was effective for some depressed patients [36]. Hypercotisolemia is detected in severely depressed patients with auditory hallucinations or delusions [37]. Glucocorticoid antagonists have been reported to have treatment effects in these patients.

Substance P—neurokinin-1 (NK1) receptor pathways have been implicated in the pathophysiology of depression. The NK1 receptors are widely expressed throughout the central nervous system and involved in regulating the stress response and controlling affective behavior, such as depression [38]. A lot of evidence that has been generated from animal experiments indicates that a NK1 receptor antagonist might be effective in the treatment of depression [39]. However, consistent results of NK1 receptor antagonism as an effective antidepressant strategy have not been reported in humans. However, NK1 receptor antagonists deserve to be placed in the spotlight. First, their chronic administration causes increased firing of serotonergic neurons [40]; Second, they induce hippocampal neurogenesis and increase the BDNF [41]. These results suggest that NK1 receptors antagonists can be usefully used as augmentation agents with a traditional antidepressant.

### 2.6. Neuroimmune and Cytokine Hypothesis

Immune responses are thought to be involved in the base mechanism of depression. Patients with immunological diseases and those who are treated with cytokines show a high frequency of depression [42]. Depressed patients without physical diseases showed an increase in inflammatory markers [43]. The symptoms of fatigue, depression, and affective flattening are notably observed in nearly 90% of hepatitis C or cancer patients who undergo IFN (interferon) treatments. With high-dose IFN-α treatment, at least 50% of patients satisfied major depressive disorder criteria within three months [44]. IFN-α administration resulted in an increase in both IL-6 and TNF-α levels, which correlated with depression severity [45].

In summary, inflammatory changes affect the signal transmission pattern of the brain, which plays an important role in depression and antidepressant effects and may be associated with neurogenesis and neural plasticity [46]. Inflammation causes or maintains depression, so inflammatory markers can be used for diagnosis, identifying treatment effects, and predicting the prognosis of depression. The effects of currently available antidepressant medications on inflammatory cytokines/mediators in depressed patients are summarized in Table 1.

**Figure 4:**
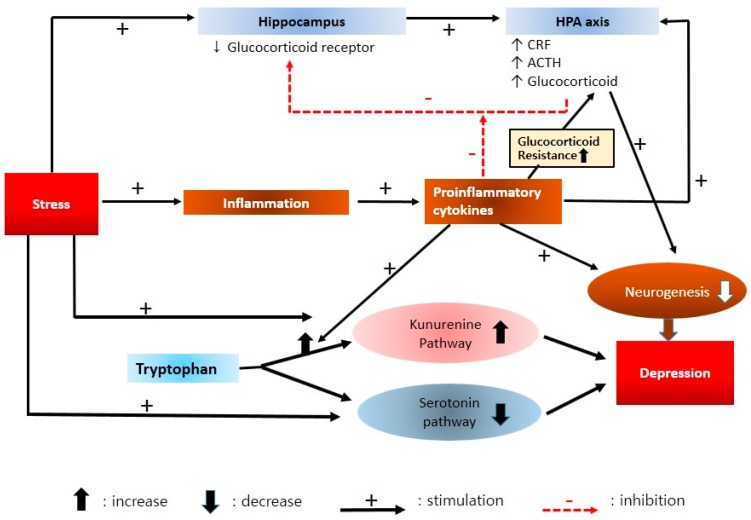
Neuroendocrine and neuroimmune interactions in depression.

According to the most recent studies, the inflammatory markers that showed a consistent increase in depression were IL-6, TNF-α, TNF-β1, IFN, and CRP [60]. These cytokines increased peripheral glucocorticoid resistance [61]. As a result, HPA axis function was weakened. These cytokines promoted transference from tryptophan (precursor of serotonin) to kynurenine rather than to serotonin [62]. As a result, the utilization rate of serotonin was decreased. The low serotonin utilization rate is assumed to be a causative mechanism of depression, as discussed in the aforementioned monoamine hypothesis (Figure 4).

Stress triggers activation of the HPA axis and the inflammatory response system. Inflammation produces activated pro-inflammatory cytokines. Pro-inflammatory cytokines elevate CRF production and interrupt negative cortisol feedback. These cytokines also increase peripheral glucocorticoid resistance. As a result, the HPA axis is weakened. These cytokines and excessive cortisol inhibit neurogenesis in the brain. The serotonin pathway shifts from tryptophan to kynurenine rather than serotonin under stress. Moreover, proinflammatory cytokines promote transference to the kynurenine pathway. As a result, the utilization rate of serotonin decreases. A low serotonin utilization rate is a causative mechanism of depression, according to the monoamine hypothesis.

Stress certainly increases cytokine levels, but the inflammatory markers extracted from the blood do not necessarily represent the status of the central nervous system. Moreover, it is not clear whether an increase in inflammation causes depression, or whether inflammation increases as a result of depression. The inflammatory response is potentially one of the broad “super-networks” of allostatic load. The inflammatory response has an immune response element amplification, which is associated with the desensitization of glucocorticoid negative feedback, a reduction in parasympathetic nerve conduction, a reduction in BDNF formation, an increase in activity in the anterior cingulated cortex, hippocampal atrophy, an increase in fat cells, and arteriosclerosis [63].

If inflammation contributes to the pathogenesis of depression, anti-inflammatory drugs may be effective for depression. Theoretically, not only typical anti-inflammatory drugs but also cytokine receptor inhibitors, cytokine antibodies and anti-inflammatory cytokines may be used as treatments for depression. Adding celecoxib, a Cyclooxygenase-2 (COX-2) inhibitor, to a typical SSRI produced more favorable results than the SSRI alone [64]. The use of celecoxib reinforcement therapy for bipolar depressive episodes enhanced treatment responses [65]. Acetylsalicylic acid (aspirin) could supplement the functions of SSRI [66]. An ω-3 fatty acid (e.g., eicosapen-tanoic acid (EPA) and docosahexaenoic acid (DHA)) also supplemented antidepressants by reducing inflammation-promoting cytokines [67]. Angiotensin receptor blocker, an antihypertensive drug, is thought to have anti-inflammatory effects on cardiovascular diseases and the central nervous system [68]. If its mechanism is clarified, it may be used for the treatment of depression.

### 2.7. Genetic Biomarkers

The candidate genes associated with major depression were discovered through collective studies on family history and twins that targeted patients with depression and patients treated by antidepressants. The recombinant frequency of inheritability is represented as the LOD (logarithm (base 10) of odds) score. The allele inheritability is calculated mainly through linkage analyses. If the LOD score is greater than 3, it is associated and comparatively inheritable. The genes associated with depression, which have been discovered through these analyses, are as follows:

(1) Tyrosin hydroxylase (*TH*) genes. Chromosome 11 has *TH* genes that express the TH enzyme, which is critically associated with dopamine synthesis. Healthy individuals suffered from depressive symptoms when TH inhibition was induced [69].
(2) Serotonin transporter (*SLC6A4*) genes. *SLC6A4* genes are located on chromosome 17. The expression of the serotonin transporter is regulated in part by an insertion/deletion polymorphism in the serotonin-transporter-linked polymorphic region (5-HTTLPR). The *SLC6A4* genes have two types of polymorphisms: the *528 (L)* allele with a long promoter region, and the *484 (S)* with a short one. In case of the *484 (S)* allele, reduced expression of the serotonin transporter was observed on the nerve cell membrane [70], and severe depression symptoms such as suicide and melancholic depression were reported [71]. When individuals with the *484 (S)* allele suffered from physical and emotional abuse during childhood, they showed a higher incidence of major depression than those without this allele [72].
(3) cAMP response element binding 1 (*CREB1*) gene. The *CREB1* gene was found on chromosome 2 of a woman who suffered from early depression and repetitive recurrence [73]. The *CREB1* gene expresses CREB1 transcription factor. CREB1 regulates the expression of growth factors such as BDNF and VEGF (vascular endothelial growth factor), which are involved in synapse development and neurogenesis [74]. When antidepressants were administered, neuronal differentiation and neurogenesis increased through the cAMP-CREB cascade [75].
(4) Piccolo presynaptic cytomatrix protein (*PCLO*) genes. *PCLO* genes express the protein Piccolo, and are associated with chromosome 7. Piccolo protein is involved in monoamine transmission, such as serotonin, epinephrine, and dopamine transmission in the brain. Among the *PCLO* genes, rs2522833 has a single nucleotide polymorphism (SNP) and is known to be associated with depression through regulation of the HPA axis [76].
(5) 5-Hydroxytryptamine (serotonin) receptor 2A (*HTR2A*) gene. The *HTR2A* gene is located on chromosome 13 and expresses the serotonin receptor. The *HTR2A* gene has almost the same functions as the serotonin transporter genes [77].
(6) *BDNF* genes. *BDNF* genes is associated with chromosome 11. Major depression is frequently observed when the *Val66Met* gene (valine being replaced with methionine) is included. Heterozygous individuals with the *Val66Met* allele showed a higher frequency of depression when they suffered from abuse during childhood, compared with homozygote individuals with *val*/*val* alleles [78].

### 2.8. Food Intake and Metabolism

The effects of food intake and metabolism on depression have been investigated. The nerve cells containing melanin concentrating hormone (MCH) stretch from the lateral hypothalamus to the limbic system, including the NAc. They mainly conduct signal transmission that promotes appetite. An overall reduction in MCH-induced signal transmission and MCH antagonism in the NAc showed antidepressant effects in rodents [79]. Thus, MCH antagonists may be helpful for depressed patients who complain of weight gain (atypical depression). In contrast to the depression-inducing effects of MCH, ghrelin can produce antidepressant effects in depressed patients with a poor appetite [80]. Metabolic status significantly affects mood and motivation in animals. Thus, understanding the correlation between peripheral metabolic signal transmission and the regulators secreted from the central nervous system, which influence food intake and wakefulness, may suggest a new perspective on depression.

Leptin, which is a satiety hormone, is secreted from white fat cells and is involved in food intake regulation. Considering that poor appetite and reduced food intake are common symptoms of depression, studies on the role of leptin in depression have been conducted. Low leptin levels were associated with depression [81], and patients who attempted suicide showed significantly low leptin levels in their cerebrospinal fluid [82]. However, other studies reported that depressed patients showed high leptin levels [83]. These high leptin levels can be explained as leptin resistance. This may be similar to insulin resistance in type 2 diabetic patients. Obese individuals have been reported to experience depression more often than normal-weight subjects [84]. High leptin levels and leptin resistance are often observed in obesity [85]. The question is whether the leptin resistance level can be a biological indicator of the coexistence of depression and obesity or not. In obese depressed patients, an intervention for leptin resistance may help.

### 2.9. Circadian Rhythms and the Melatonergic System

A disorganization of circadian rhythms has been suggested to play an important role in the pathophysiology of major depression [86]. The circadian rhythms are associated with the nocturnal rise of endogenous melatonin secretion. Depressed patients exhibit disturbance in the melatonin secretion rhythm, and the quality of sleep in these patients can be improved by the melatonin [87]. Melatonin secretion is controlled by the suprachiasmatic nucleus (SCN) and high concentrations of melatonin receptor occur in the SCN. Because of melatonin’s short half-life, its suitability as a drug is limited [88]. Thus, the development of prolonged-release melatonin and other melatonergic receptor agonists with a longer duration of action were needed.

Agomelatine is an emerging antidepressant, with a unique profile of selective antagonist at 5-HT2C receptors and melatonin receptor agonist (MT1/MT2) [89]. Previous studies have shown its superior efficacy to placebo in treating major depression. Previous trials have shown comparable antidepressant efficacy of agomelatine compared to monoamine-series antidepressants [87,90]. Agomelatine does not include 5-HT2A stimulation. Therefore, it seems to be not associated with sexual dysfunction and it has less potential for serotonin syndrome or discontinuation syndrome than standard antidepressants including SSRI. In addition, agomelatine is able to enhance neuroplasticity mechanisms and adult neurogenesis in brain area such as hippocampus and prefrontal cortex [87]. Considering favorable results on the efficacy and safety of agomelatine in treating depression, it could be a good and safe treatment alternative in the treatment of depression.

### 2.10. New Insight to Protein Translation Signal (mTOR) Pathways

Ketamine (a non-competitive NMDA glutamate receptor antagonist) has antidepressant activity [3]. The activation of protein-translation signaling pathways plays a key role in the long-term regulation of neural circuits. Active protein translation processes are required for development, differentiation, growth, synapse formation, nervous branch formation, and axonal growth-differentiation in neural cells [91].

The protein translation process is regulated by a dynamically-reciprocal signal transmission system [92]. The initiation stage of protein translation is a critical state for regulation, with MAPK (mitogen-activated protein kinase) and Akt (protein Kinase B) serving as representative modulators. Akt phosphorylates mTOR (mammalian target of rapamycin), and this induces phosphorylation of 4E-BP (eukaryotic translation initiation factor 4E binding protein) and p70S6K (p70 ribosomal S6 kinase). ERK1/2 (extracellular-signal-regulated kinase) can also induce phosphorylation of p70S6K. Phosphorylation-activated p70S6K then phosphorylates S6 protein (small ribosomal S6) and eIF4B (eukaryotic translation initiation factor 4B). p90 ribosomal s6 kinase (p90RSK), which is regulated by ERK1/2, can also independently induce S6 to be active. ERK1/2 and Akt reciprocally regulate the initiation stage of protein translation (Figure 2).

NMDA receptor antagonists such as ketamine, phencyclidine, and MK-801 (dizocilpine) cause dissociated anesthesia and can induce psychotic symptoms [93]. NMDA receptor antagonists protect nerve cells and inhibit nerve cell damage caused by an increase in NMDA receptor hyperactivity and calcium influx [94]. The long-suspected antidepressant effects induced by NMDA receptor have been re-explored in most recent mechanistic, structuralized studies. A ketamine injection in treatment-resistant patients with depression induced very rapid antidepressant effects [95].

The protein translation process plays an important role in the rapid antidepressant effects of NMDA receptor antagonists. ERK1/2- and Akt-mediated mTOR signal transmission was activated by ketamine, showing rapid antidepressant effects. Consequently, synapse formation in the frontal lobe increased. An mTOR antagonist inhibited the antidepressant effects of ketamine and synapse formation. These results suggest that mTOR plays a major role in antidepressant effects through ketamine [96]. Ketamine increased BDNF protein translation by inhibiting the activity of eukaryotic elongation factor 2 (eEF2) kinase. The inhibition of eEF2 kinase itself induced rapid antidepressant effects. This confirmed the role of eEF2 kinase in the antidepressant effects of NMDA receptor antagonists [97]. In addition, NMDA receptor antagonists improved depression-related behaviors and inhibited synapse reduction caused by chronic stress [98]. Molecular expression changes of mTOR in the posthumous brain of depressed patients were also reported [99].

In summary, ERK1/2 and Akt play significant roles in the pathogenesis of depression. The roles of mTOR and eEF2 kinase are important in the rapid antidepressant mechanism of NMDA receptor antagonist. In depression, the significant neural role of protein translation signaling pathways may provide targets for new antidepressants.

## 3. Materials and Methods

The MEDLINE/PubMed, EMBASE, and ClinicalTrials.gov databases were searched from inception through December 2015 for published randomized-controlled trials, open label trials, meta-analyses and systematic reviews for neurobiology and novel targets of depression. Searches included various combinations of the following terms: depression, major depressive disorder (MDD), neurobiology, molecular, cell, receptor, novel targets, novel treatment, antidepressant, biomarker, neuroanatomy, neural circuitry, monoamine, neural plasticity, neurogenesis, neurotrophin, TrkB, brain-derived neurotrophic factor (BDNF), endocrine, hypothalamic-pituitary-adrenal (HPA) axis, glucocorticoid, immune, cytokine, inflammation, genetic, gene, metabolism, leptin, melatonin, agomelatine, mTOR, glutamate, and ketamine. Ongoing clinical trials for new treatment in depression were also searched for on ClinicalTrials.gov. Studies were limited to those that assessed human subjects and English-language literature.

First, the titles of all the results were read to select articles which are relevant to the topic of this review. Second, the abstracts of the selected articles were reviewed to further identify articles that were appropriate, and the full texts of these articles were collected for review. Third, more recent studies (within the last decade) were prioritized and overlapped study designs were excluded. The references of published articles identified in the initial search process were also examined for any additional studies appropriate for the review. Finally, 59 articles were selected as being relevant to the present review.

## 4. Conclusions

Our review is narrative and this is an inherent limitation to our work. Although the authors carefully selected the papers according to a search strategy, these papers may reflect the authors’ preference. Our topic are excessively broad and heterogeneous, and samples were studied using different measurements, time points, and variable patients. However, every effort was made to summarize available evidence from preclinical studies as well as from both published and ongoing trials.

Theories regarding the pathophysiology of depression began with the excessive black bile theory (Cladius Galen, 130–200 A.D.) and developed into psychological shocks and neurotransmitter imbalances. Most recently, endocrinological mechanisms, immunological mechanisms, cells and molecules, metabolic regulation, and genetic-environmental factors have been suggested. Nevertheless, there is a difference between understanding depression and treating it. Researchers and clinicians should not be intimidated by the diverse pathogenesis of depression, but should continue to clarify the neurobiological mechanisms of depression based on its various symptomatic features.

Future studies should target specific brain regions associated with depression. Comprehensive and multilateral studies may be required to clarify the causative relationships between depressive symptoms and the neuronal network, including cytokines, neurotransmitters, and neurohormones. Eventually, the genes that induce depression must be verified to understand interactions between the genes and the environment. Recently, studies on neural plasticity and mTOR signal transmission have been conducted. Based on the outcome of these studies, the molecular and cellular mechanisms of depression should be clarified to facilitate neurobiological approaches. Integrated studies on genes, proteins, and animal behavior may help to build a clearer understanding of the pathogenesis and treatment mechanisms of depression, and may enable new drug development and clinical applications.

The limited effects of current antidepressants constitute an urgent issue in depression studies. Non-monoamine-series antidepressants are expected to be developed to solve this problem, but efforts regarding development have not yielded satisfactory results over the past several decades. Nonetheless, the failures of past studies will pave the way for successful future research.

## Figures and Tables

**Table 1:** Serum and plasma levels of cytokine in depressed patients before and after antidepressant treatment.

Study	Antidepressant (Dose (mg))	Period (Weeks)	Cytokine Production
Maes *et al.*, (1995) [47]	Fluoxetine (>20 mg) and TCA (NS)	12	↔IL-6, ↔sIL-6R, ↔sIL-2R
Sluzewska *et al.*, (1995) [48]	Fluoxetine (NS)	NS	↓IL-6
Frommberger *et al.*, (1997) [49]	NS (NS)	8	↓IL-6
Basterzi *et al.*, (2005) [50]	SSRI (NS)	6	↓IL-6
Maes *et al.*, (1997) [51]	Fluoxetine (20 mg), Trazodone (100 mg)	5	↔IL-1Ra, ↔IL-6, ↓IL-6R
Hinze-Selch *et al.*, (2000) [52]	TCA (NS)	6	↑sTNF-αRI
	Paroxetine (NS)	6	↔sTNF-α RI, ↔TNF-α , ↔sTNF-α RI, ↔sIL-2R
Himmerich *et al.*, (2006) [53]	Various antidepressant (NS)	NS	↔TNF-α, ↔sTNF-αRI, ↔sTNF-αRII
Tuglu *et al.*, (2003) [54]	SSRI	NS	↓TNF-α
Kagaya *et al.*, (2001) [55]	Clomipramine (NS)	4	↔IL-1β, ↔sIL-2R, ↔IL-6, ↑TNF-α
Mikova *et al.*, (2001) [56]	Various antidepressant (NS)	6	↔sIL-2R, ↔IL-6, ↔IL8, ↔ TNF-α
Kim *et al.*, (2002) [57]	Various antidepressant	8	↓IL-12
Lee and Kim (2006) [58]	Various antidepressant	6	↓IL-12, ↑TGF-β
Sutcigil *et al.*, (2007) [59]	Sertraline (50–100 mg)	8	↓IL-2, ↑IL-4, ↓IL-12, ↓TNF- α, ↑TGF-β

TCA: tetra-cyclic antidepressant, SSRI: selective serotonin reuptake inhibitors, ↑: increase, ↓: decrease, ↔: no change of cytokine level, NS: not specified.
